# Prediction of enzyme function based on 3D templates of evolutionarily important amino acids

**DOI:** 10.1186/1471-2105-9-17

**Published:** 2008-01-11

**Authors:** David M Kristensen, R Matthew Ward, Andreas Martin Lisewski, Serkan Erdin, Brian Y Chen, Viacheslav Y Fofanov, Marek Kimmel, Lydia E Kavraki, Olivier Lichtarge

**Affiliations:** 1 Department of Molecular and Human Genetics, Biophysics, Baylor College of Medicine, Houston, TX 77030, USA; 2 Graduate Program in Structural and Computational Biology and Molecular Biophysics, Baylor College of Medicine, Houston, TX 77030, USA; 3 Department of Computer Science, Rice University, Houston, Texas 77030, USA; 4 Department of Statistics, Rice University, Houston, Texas 77030, USA; 5 Department of Bioengineering, Rice University, Houston, Texas 77030, USA

## Abstract

**Background:**

Structural genomics projects such as the Protein Structure Initiative (PSI) yield many new structures, but often these have no known molecular functions. One approach to recover this information is to use *3D templates *– structure-function motifs that consist of a few functionally critical amino acids and may suggest functional similarity when geometrically matched to other structures. Since experimentally determined functional sites are not common enough to define 3D templates on a large scale, this work tests a computational strategy to select relevant residues for 3D templates.

**Results:**

Based on evolutionary information and heuristics, an Evolutionary Trace Annotation (ETA) pipeline built templates for 98 enzymes, half taken from the PSI, and sought matches in a non-redundant structure database. On average each template matched 2.7 distinct proteins, of which 2.0 share the first three Enzyme Commission digits as the template's enzyme of origin. In many cases (61%) a single most likely function could be predicted as the annotation with the most matches, and in these cases such a plurality vote identified the correct function with 87% accuracy. ETA was also found to be complementary to sequence homology-based annotations. When matches are required to both geometrically match the 3D template and to be sequence homologs found by BLAST or PSI-BLAST, the annotation accuracy is greater than either method alone, especially in the region of lower sequence identity where homology-based annotations are least reliable.

**Conclusion:**

These data suggest that knowledge of evolutionarily important residues improves functional annotation among distant enzyme homologs. Since, unlike other 3D template approaches, the ETA method bypasses the need for experimental knowledge of the catalytic mechanism, it should prove a useful, large scale, and general adjunct to combine with other methods to decipher protein function in the structural proteome.

## Background

Structural genomics projects such as the Protein Structure Initiative ("PSI") aim to provide an experimental structure for all proteins [1-3], but as of May 2007 over one third of the nearly 4,400 protein structures deposited into the protein structure databank (PDB) [4] with the keyword "structural genomics" were either hypothetical proteins or without known function. The annotation of these structures remains an important goal essential to understanding their biological meaning. Ideally, such annotations might be obtained experimentally, through automated generalized screens for some enzymes [5]. However, further efforts are required to develop efficient and large scale assays that cover the most relevant protein functions, and so far just 5% of current annotations are from direct experiments [6].

Thus 95% of annotations rely on the computational identification of similarity between a protein of unknown function and one of known function. Most frequently this similarity is common ancestry, identified by BLAST [7] or PSI-BLAST [8]. This is most reliable when sequence identity is above 40% and matched with a profile [9-11], but errors will occur at lower sequence identity [9,12-16]. Probably some fraction of annotations are misleading [17,18] and may even propagate to other proteins [19-21]. It is thus imperative to develop and combine new techniques to increase annotation reliability. Meta-servers such as ProFunc [22] and JAFA [23] pool annotations from multiple sources. In order to raise the predictive value of these servers it is important to continue to improve each individual method.

These other methods exploit other types of functionally relevant similarities between proteins: one such is general structural similarity (DALI [24], VAST [25], SSM [26], Grath [27], PDBFun [28], TOPS [29], SuMo [30,31], CM [32]); another is local sequence similarity of a few residues that are highly specific to function, such as pre-defined sequence motifs [33,34]. Such motifs can be generalized to structures as 3D templates that represent key functional residues and their geometry. Examples include the geometric matching of 3D templates to proteins of unknown function (Jess [35,36], Rigor [37], Pints [38], ASSAM [39], GASPS [40], and several methods used by ProFunc [22,41]), the comparison of surface patches or clefts (Surfnet [42], VOIDOO [43], CASTp [36], SiteEngine [44], pvSOAR [45]), or of structural binding site locations (Surfnet-ConSurf [46], eF-site [47], Cavbase [48], PDBSiteScan [49,50]). Such 3D templates may identify functional analogs that converged to perform the same function despite sharing no discernable homology [51]. Overall, though, the total number of motifs that are experimentally identified remains small compared to the vast number and functional diversity of proteins [52].

The goal of this study is to generalize 3D template annotation methods by addressing the limited number of experimentally determined templates. Our hypothesis is that even without prior knowledge of a catalytic mechanism, evolutionary information can suffice to identify functional sites, extract representative 3D templates and search for relevant geometric matches in other structures. To test this possibility, we use the Evolutionary Trace (ET) [53-57]and build an automated Evolutionary Trace Annotation (ETA) pipeline. Benchmarks on 98 enzymes show that the annotation accuracy of ETA is high and remains so at low sequence identity, making it a useful complement to homology annotations.

## Results and Discussion

### Annotation Pipeline Overview

**Figure 1:**
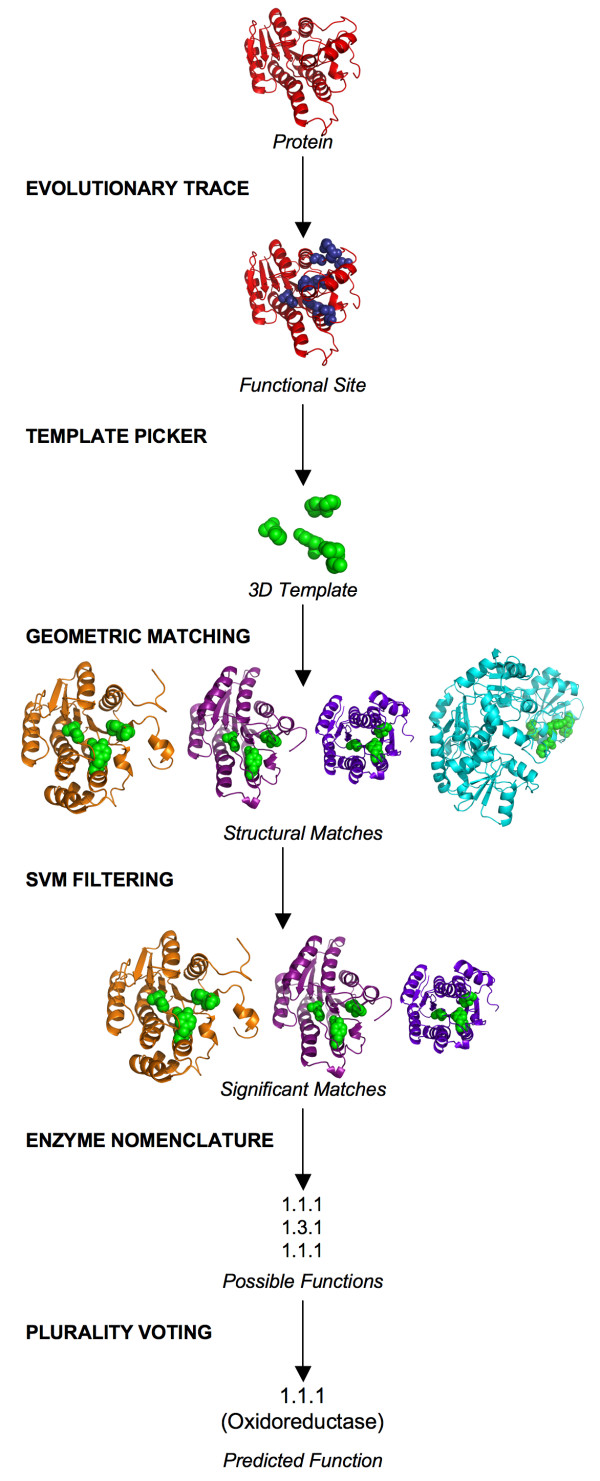
Illustration of the automated functional annotation pipeline.

The ETA functional annotation pipeline integrates the steps in Figure 1. The input, or *query X*, is a protein structure of unknown function. Step one constructs a *3D template*: points in a precise relative geometry that represent the locations and types of amino acids deemed necessary and sufficient for the activity of *X*. Next, the 3D template is matched in other structures from the PDB, or *targets*, to identify those with similar local structures – meaning that the 3D template can be superimposed to closely match some part of the target. To further increase functional relevance, a filter only accepts matches that fall on evolutionarily important sites in the target. The function of *X *is then predicted to be one of the functions among the remaining matches, and specifically to be the one found most often if such a *plurality *exists.

Each of these tasks is broken down into smaller steps. To build templates, the method first uses ET to rank the residues of a protein structure by their relative evolutionary importance [56] and to locate a functional site by identifying the largest structural cluster of top-ranked residues. A *template picker *routine then uses heuristics to select specific residues and choose points to represent them (see below). To search for local structural similarity between a template and a structure in the PDB, the Match Augmentation (MA) algorithm [58,59] searches for sub-structures with geometric and chemical similarity to the template residues and organizing the search by ET ranks. Next, a geometric filter selects the most statistically significant matches based on the least root-mean-squared-deviation (RMSD) relative to the template, and a support vector machine (SVM) further selects matches based on the evolutionary importance and geometric similarity of the matched residues. The Enzyme Commission annotations (EC numbers) [60] of these significant matches represent a set of possible functions for the query protein. Since spurious matches should involve random functions, we hypothesize that a function with the most matches – the one with a plurality of matches – is most likely to be accurate.

### Template design without prior knowledge of functional sites

**Figure 2:**
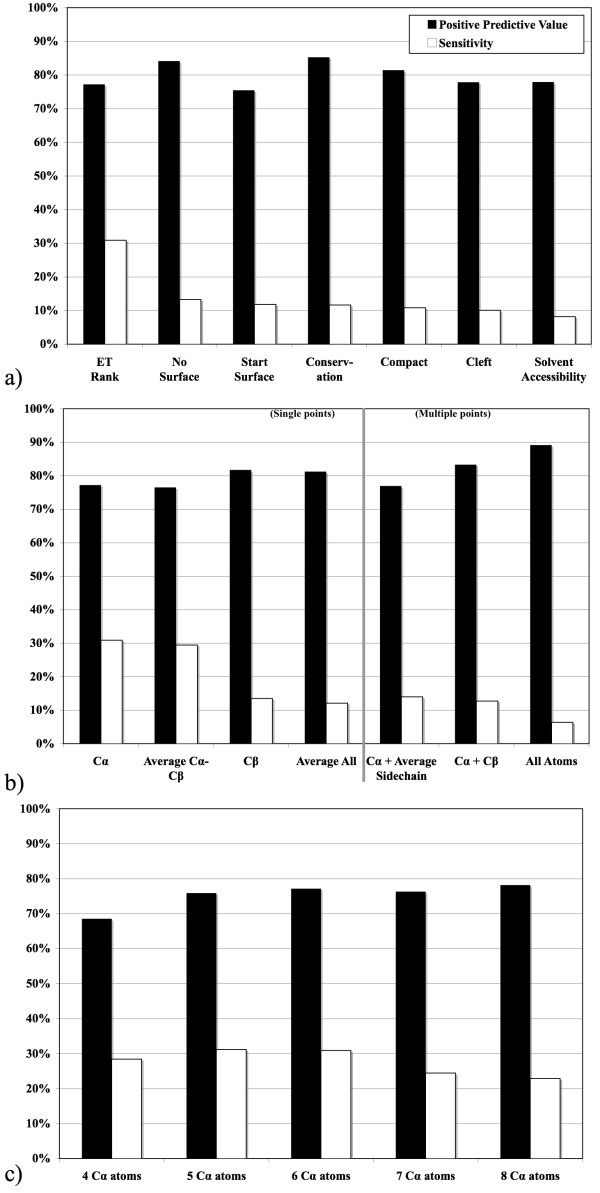
Average positive predictive value (black bars) and sensitivity (white bars) in the training set for several heuristics: (a) choosing residues for the 3D template; (b) representing those residues as points, with single-point methods left of the grey line and multiple-point methods right of it; or (c) choosing the size of the template.

In order to design 3D templates we tested several heuristics to choose how many residues to include; which ones to pick; and how to represent them geometrically. In turn, several heuristics for one of these choices were tested in a training set of 53 diverse enzymes while the other two choices were held at reasonable values. For example, to select which residues to pick, the template size was set at six residues and the template representation was set to C_α _atoms only. Then, starting from a protein surface cluster of at least 10 top-ranked ET residues, alternative templates were constructed based on heuristics that biased templates towards ET rank, sequence conservation, solvent accessibility, or local topology (see Methods). These templates were then processed in the ETA pipeline and, as shown in Figure 2(a), the ET Rank heuristic, which picks the most evolutionarily important residues, had the best positive predictive value (PPV) of about 80% – in line with other choices – but a 2- to 3-fold improvement in sensitivity over the other heuristics.

To choose the best geometric representation, we considered either one or many points per template residue, centered on atoms or pseudo-atoms. A simple backbone C_α _representation was chosen given its 2- to 3-fold higher sensitivity with little decrease in PPV compared to other methods (see Figure 2b). Finally, after testing template sizes between 4 and 8 residues per template we chose 6, because it yields the best combination of PPV and sensitivity (Figure 2c). In summary, reasonable 3D templates appear to consist of the six most evolutionarily important residues from a surface trace cluster represented them by their C_α _atoms.

### Functional relevance and optimality of templates

The rationale for building templates from clusters of top-ranked ET residues is that the latter usually overlap functional sites [55]. In order to confirm the functional relevance of the automated 3D templates, they were therefore compared with SITE records (functional site residues identified in PDB structure files [4]) and CSA (Catalytic Site Atlas) records [52]. Consistent with the lack of experimentally identified functional sites, only 10 and 33 of the 53 training set enzymes had SITE and CSA records, respectively. Of these, half (5 and 18 respectively) consisted of only two residues, and only two in each case had six or more residues.

**Figure 3:**
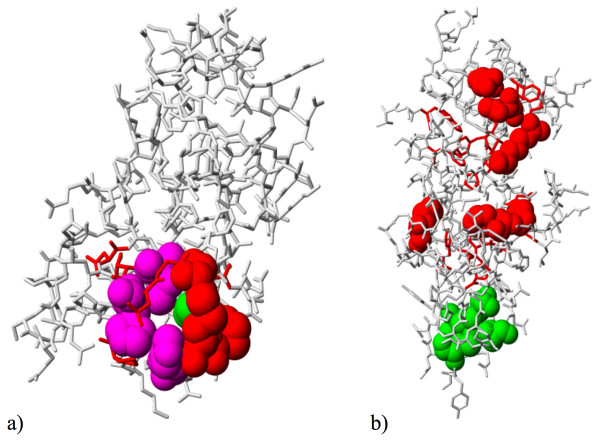
Overlap (purple spheres) of ET Rank template residues (red spheres) with SITE records (green spheres) provided by the PDB, in the context of the surface trace cluster (red sticks) from which the template residues were chosen. (a) Rieske iron-sulfur protein (PDB 1RIE); (b) Casein kinase II (PDB 1QF8, chain A).

The ET-based templates fully identified the SITE residues once, and partially overlapped them in six other cases, as shown for the Rieske iron-sulfur protein shown in Figure 3(a). In the three remaining cases, the SITE record was not matched yet the templates were still biologically relevant. In casein kinase II (Figure 3(b)), the SITE records describe only one of two metal-binding sites (Zn and Mg), while the template picker identified the other one. In beta-lactamase, there are three ligands, and the ETA template surrounding the two non-metal ligands is not described in the SITE records. In the final example, the template overlaps with records in the CSA [52]. Thus the templates are functionally relevant in all 10 cases, although neither they nor the SITE records are a complete representation of the proteins' functional sites.

Likewise, comparison with CSA shows the ET-based templates typically overlap the known functional sites. Of the 33 proteins with CSA records (89 residues total), 24 (73%) proteins include one of these residues in the template itself, and 26 proteins (79%) found at least one of these residues at the same rank that the template was picked from. Furthermore, all but one of the templates were chosen from the same region as the CSA templates. In the single exception, 1goiA, the template residues were picked from a highly-ranked arm of the protein whereas the CSA annotations form a cluster of less highly-ranked residues in a cleft near the center.

**Figure 4:**
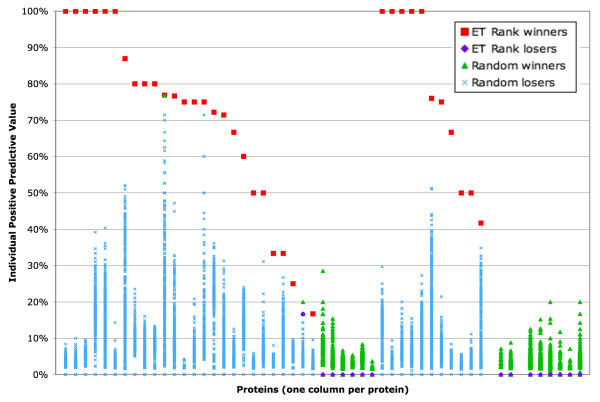
PPV for each of the 53 proteins in the training set (highest PPV in red squares, others in purple diamonds) and for the templates with randomly chosen residues (green triangles, highest PPV; blue 'X's, others). All residue combinations were sampled for the 32 proteins on the left; 500 templates were randomly sampled for the 21 proteins on the right.

Although ETA templates are functionally relevant, they may not be optimal. To test this possibility we compared them, in the training set, to a random sample of up to 500 unique, six residue, C_α_-only templates randomly sampled from surface ET clusters. As shown in Figure 4, the PPV of ETA templates is best in 35 of the 53 proteins, often by a wide margin. In 32 proteins, the surface trace cluster had only 11 residues, so all 462 possible choices were tested. ETA templates had the best PPV in 24 of these 32 cases, and the second best one in 2 more. In 16 cases where ETA templates do not perform well, none of the random templates achieves a large PPV. Overall, ETA templates are close to optimal, suggesting that they typically do capture a small number of top-ranked surface residues that are functionally informative and yield high PPV.

### Template Matching

**Figure 5:**
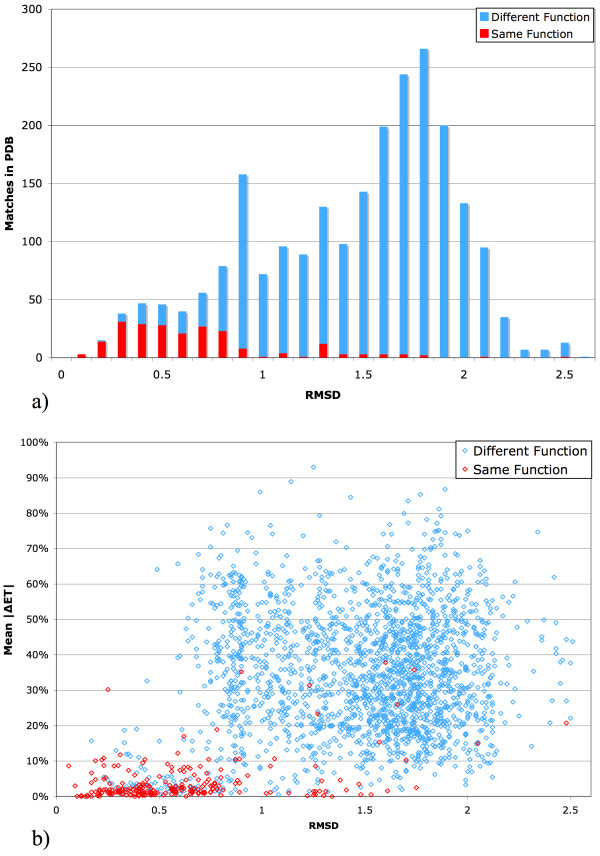
(a) Stacked cumulative histogram of RMSDs of significant (p-value ≤ 1%) matches, including matches with the same function (red) and different function (blue). (b) Scatterplot of these same matches, adding the average absolute value of the difference in evolutionary importance between the matched and query residues to allow separation of true and false matches.

The next step is to identify template matches. A search against the PDB finds on the order of 5,000 matches that fit the template's amino acids and geometry over a wide range of RMSDs, from 0–10Å, and a statistical filter then retains only those with p-values below 1%. Most of these remaining matches are still functionally irrelevant, as seen in Figure 5(a), because a single p-value cannot be biologically relevant in all cases: Some, like kinases or proteases, will have very many functional homologs and analogs while other proteins will have very few.

The evolutionary importance of the matched target residues is an essential criterion to distinguish true from irrelevant matches [56]. The rationale is that biologically relevant matches should fall on evolutionarily important sites while a random match will fall anywhere, regardless of evolutionary importance. Figure 5(b) shows that indeed the ET ranks of target residues separate many false matches. Once an SVM was trained (see Methods) to separate matched targets based on RMSD and evolutionary importance, it considerably narrowed the range of possible annotations as only a handful of matches are left.

### Functional Annotation

The ETA pipeline was tested first in 49 enzymes with known EC numbers picked randomly from the PDB (see Methods). ETA extracted templates from each, matched them in the PDB90 (see Methods), and extracted targets accepted by the SVM. Matches to target proteins with the same first 3 EC number digits as the query protein were deemed correct. On average each template had 3.3 matches, and 2.1 were correct. The range was variable, however: 10 templates had no matches at all, while two templates had 34 hits and 10 hits, respectively. As described in Table 1, 33 of the 39 templates match at least one protein with the correct identical function (85%). Strikingly, 32 templates achieve a plurality of matches to a single EC annotation and 30 of these identify the correct function (94%).

**Table 1:** PDB set annotation performance with all matches

	ETA	BLAST	PSI-BLAST	BLAST + ETA	PSI-BLAST + ETA	PEAS (voting)	PEAS (scoring)	PRT (voting)	PRT (scoring)
Matches	164	292	365	122	134	18	18	211	49
True matches	102	186	225	93	101	13	13	53	21
Proteins	49	49	49	49	49	49	49	49	49
With matches	39	44	46	36	36	12	12	49	49
With at least one true match	33	39	40	33	33	9	9	30	21
With vote winners	32	40	39	33	33	10	12	30	49
With correct winners	30	39	37	33	33	7	8	28	21
Prediction accuracy	94%	98%	95%	100%	100%	70%	67%	93%	43%
Prediction availability	65%	82%	80%	67%	67%	20%	24%	61%	100%

PDB set annotation performance from the methods: ETA, BLAST, PSI-BLAST, the BLAST+ETA, PSI-BLAST+ETA, and PEAS using either voting or scoring to pick the most likely function. Prediction accuracy and prediction availability refer to the voting procedure: prediction accuracy = true predictions (i.e., correct vote winners)/total predictions (i.e., vote winners), while prediction availability = total predictions (vote winners)/total number of proteins in that set.

To test ETA in the most stringent and relevant case, these retrospective control experiments were repeated in 49 randomly chosen PSI enzymes with known EC numbers, the PSI test set (Table 2). Again, the templates averaged 3.3 matches, of which 2.1 were correct. Of 38 templates with at least one match, 30 find at least one correct hit (79%). Among the 28 templates that achieve a plurality of hits to one function, that function is correct in 22 cases (79%).

**Table 2:** PSI set annotation performance with all matches

	ETA	BLAST	PSI-BLAST	BLAST + ETA	PSI-BLAST + ETA	PEAS (voting)	PEAS (scoring)	PRT (voting)	PRT (scoring)
Matches	163	177	243	107	117	10	10	75	42
True matches	93	103	120	87	90	6	6	44	27
Proteins	49	49	49	49	49	49	49	49	49
With matches	38	37	35	31	31	7	7	42	42
With at least one true match	30	31	31	28	28	5	5	30	27
With vote winners	28	30	29	25	25	6	7	29	42
With correct winners	22	24	24	24	24	4	5	26	27
Prediction accuracy	79%	80%	83%	96%	96%	67%	71%	90%	64%
Prediction availability	57%	61%	59%	51%	51%	12%	14%	59%	86%

The ultimate goal of ETA is to provide functional annotations for PSI proteins, which are more stringent than most PDB proteins because they are required to have less than 30% sequence identity with any other PDB protein [61-66]. Since these proteins represent a sparser sampling of structures than the PDB set, a concern is that the plurality voting procedure, which relies on multiple matches to the same function, will yield far fewer correct predictions in such cases, and indeed accuracy is reduced from 94% in the PDB set to 79% in the PSI set and availability is reduced from 65% to 57%. To more fully account for the effects of this structural sampling bias present in the target dataset, we removed matches with progressively decreasing levels of sequence identity to other matches by the same template in the PSI set [see Additional file 1, Table S1]. While this does not remove most of the biases inherent in the PDB (towards small, globular, easily-crystallizable proteins, for instance), it at least accounts for proteins that are more highly represented – a problem that is not completely alleviated by use of the PDB90. At 80% or 60% sequence identity, no changes are observed; however at 40% there are 2 fewer correct predictions and 1 fewer incorrect prediction, which provides similar prediction accuracy (80%) and only slightly lower availability (51%). Even at the extremely low level of 15% sequence identity, prediction accuracy only decreases to 73% and availability to 53%, indicating that the accuracy and availability of ETA and the plurality voting procedure remains high even when structural sampling is very low, as it is with most PSI proteins.

### Complementarity with homology information

**Figure 6:**
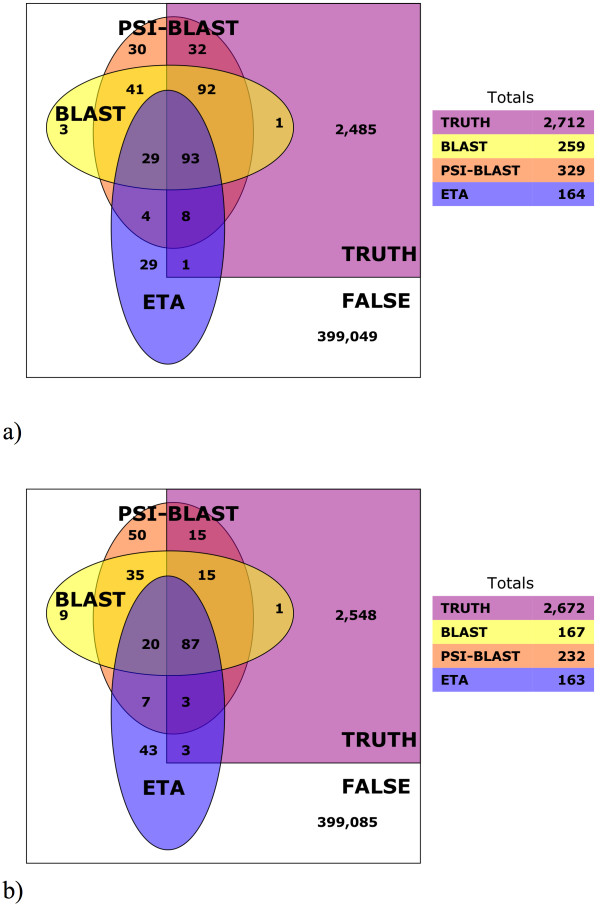
Venn diagram showing the overlap with true (purple) and false (white) matches to a) the PDB set and b) the PSI set, found by ETA (blue), BLAST (yellow), and PSI-BLAST (orange). Sum of all matches found in each category are at right.

In order to understand the nature of ETA annotations, they were compared with BLAST and PSI-BLAST, and Figure 6 suggests that most ETA matches are homologs. ETA, however, produces fewer false positives so that the specificity of functional annotation is increased by combining methods. In the first test set (Figure 6(a)), BLAST finds 186 true matches and 73 false ones, its positive predictive value, PPV_BLAST_, is thus 72%. The intersection of BLAST with ETA has fewer true matches (93) but even fewer false ones (29) so the combined PPV_ETA+BLAST _is 76% – a relative increase of 6% over PPV_BLAST_. Similarly, PPV_PSI-BLAST _is 68%, whereas PPV_ETA+PSI-BLAST _is 75%, a relative increase of 10%.

The complementarity of ETA and homology methods is even greater in the PSI set (Figure 6(b)). PPV_BLAST _is 62% whereas PPV_ETA+BLAST _is 82% – a 32% relative increase – and PPV_PSI-BLAST _is 52%, whereas PPV_ETA+PSI-BLAST _is 77%, a relative increase of 48%. Thus, ETA is complementary to homology-based annotation and substantially increases the predictive value of either BLAST or PSI-BLAST when combined with them.

**Figure 7:**
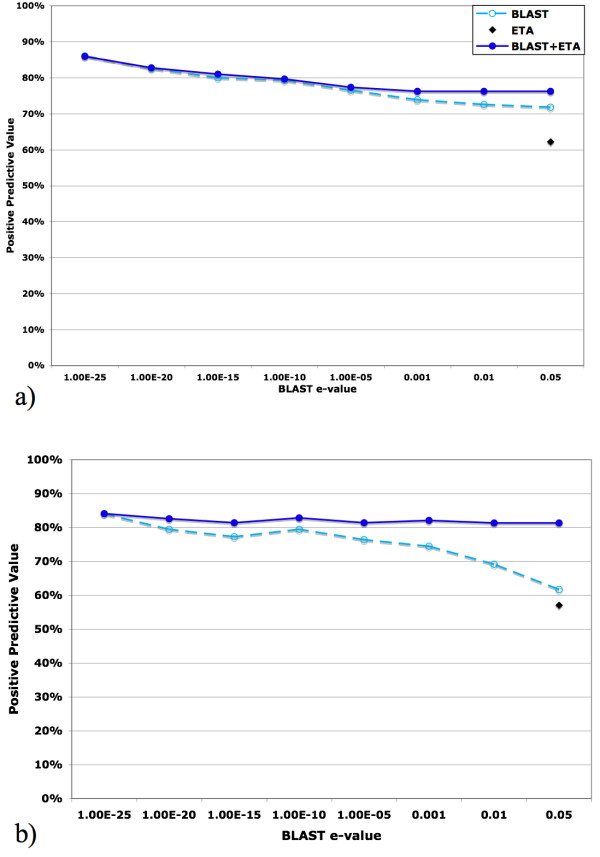
PPV of BLAST (cyan dashed hollow circle) and BLAST+ETA (blue solid circle) as the maximum e-value cutoff for BLAST varies (horizontal axis). ETA shown as a single point (black diamond) at e-value = 0.05. (a) PDB set; (b) PSI set.

One possible concern is that the increased in PPV from ETA is not significant because it can be achieved with BLAST alone simply with a more stringent e-value threshold. But, Figure 7 shows that the PPV improvement from ETA persists across a wide range of e-values and when, eventually, PPV_BLAST _and PPV_ETA+BLAST _do converge, at an e-value of 1e-25, this is at the price of much reduced sensitivity. Otherwise, for higher BLAST e-values, ETA significantly increases in PPV – especially in the PSI set where homologous structures are fewer. For example, at an e-value of 0.05, the combined method shows a 32% relative increase in PPV above that of BLAST alone.

**Figure 8:**
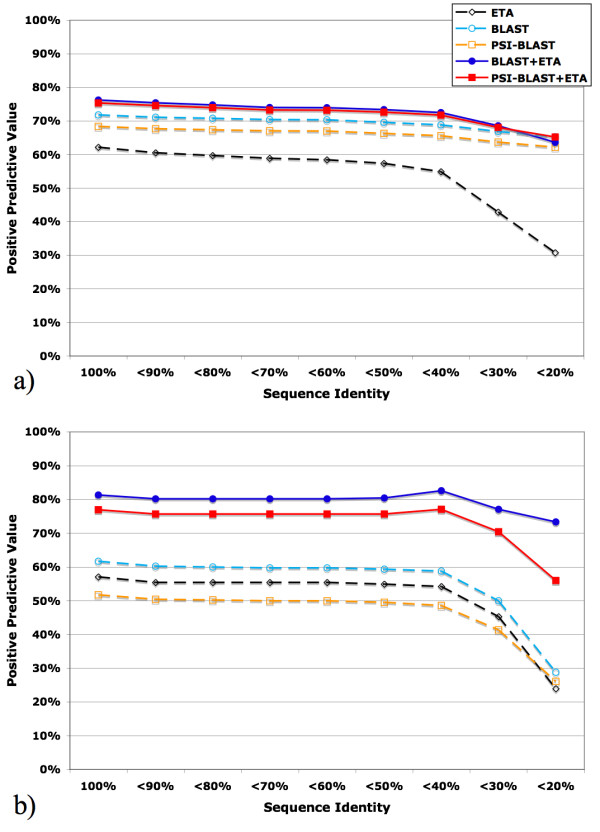
Match PPV of ETA (black dashed hollow diamond), BLAST (cyan dashed hollow circle), PSI-BLAST (orange dashed hollow square), the intersection of BLAST+ETA (blue solid circle), and the intersection of PSI-BLAST+ETA (red solid square). The horizontal axis represents decreasing levels of match sequence identity. (a) PDB set; (b) PSI set.

A second and related concern is that ETA matches might mostly arise among proteins with high sequence identity – a trivial result. Even if this were the case, ETA would still be of some use to identify residues likely to be the most functionally important in the target protein and add structural and functional evidence to that provided by sequence; however, Tables 3 and 4 suggest that these concerns are unfounded. When matches with sequence identity above a given threshold are removed, the gains in PPV from adding ETA to BLAST, or PSI-BLAST, persist as shown by Figures 8(a) and 8(b). The PPV advantage of the combined methods is about 50% greater in either the PDB or PSI test sets. For example, in the latter and at less than 20% sequence identity, PPV_BLAST+ETA _is 72% greater than PPV_BLAST_, and PPV_PSI-BLAST+ETA _is 68% greater than PPV_PSI-BLAST _alone. Thus, combining local structural matches of ETA templates with homology considerably improves functional annotation specificity, and does so most at low sequence identity.

**Table 3:** PDB set annotation performance with matches having ≤20% sequence identity

	ETA	BLAST	PSI-BLAST	BLAST + ETA	PSI-BLAST + ETA	PEAS (voting)	PEAS (scoring)
Matches	55	119	191	13	25	9	9
True matches	18	73	113	9	17	6	6
Proteins	49	49	49	49	49	49	49
With matches	24	26	33	11	16	5	5
With at least one true match	10	16	20	7	10	3	3
With vote winners	13	17	25	7	12	5	5
With correct winners	8	15	19	7	10	3	2
Prediction accuracy	62%	88%	76%	100%	83%	60%	40%
Prediction availability	27%	35%	51%	14%	24%	10%	10%

**Table 4:** PSI set annotation performance with matches having <20% sequence identity.

	ETA	BLAST	PSI-BLAST	BLAST + ETA	PSI-BLAST + ETA	PEAS (voting)	PEAS (scoring)
Matches	71	59	123	15	25	4	4
True matches	17	15	31	11	14	1	1
Proteins	49	49	49	49	49	49	49
With matches	23	22	22	9	9	4	4
With at least one true match	9	8	14	6	7	1	1
With vote winners	18	17	18	9	8	4	4
With correct winners	7	8	11	6	6	2	2
Prediction accuracy	39%	47%	61%	67%	75%	50%	50%
Prediction availability	37%	35%	37%	18%	16%	8%	8%

**Figure 9:**
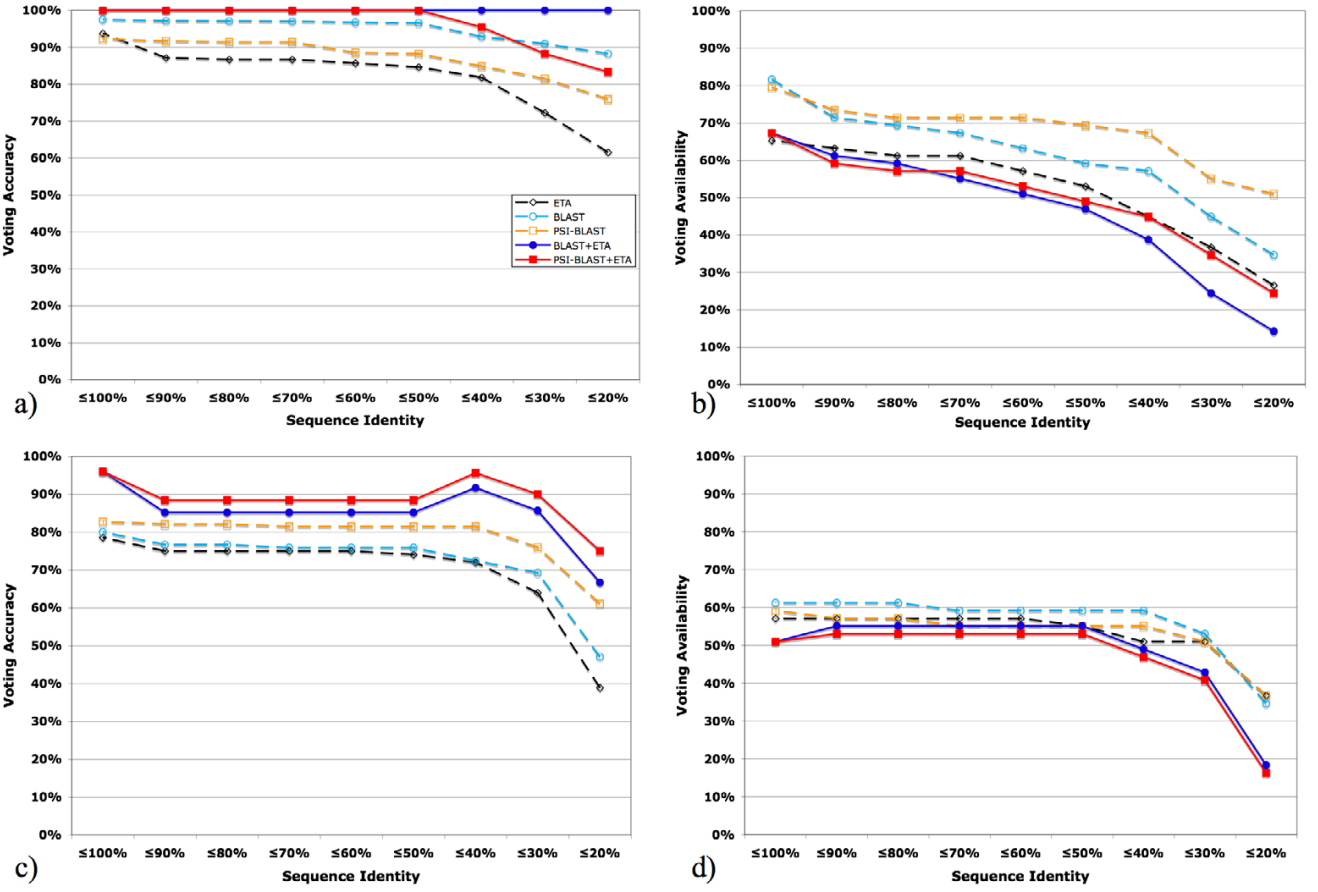
Annotation performance of ETA (black dashed hollow diamond), BLAST (cyan dashed hollow circle), PSI-BLAST (orange dashed hollow square), the intersection of BLAST+ETA (blue solid circle), and the intersection of PSI-BLAST+ETA (red solid square). The horizontal axis represents decreasing levels of match sequence identity, and the vertical axis represents: (a) PDB set voting accuracy; (b) PDB set voting availability; (c) PSI set accuracy; (d) PSI set availability.

Finally, we asked whether these increases in PPV, measured over all templates and their matches, translate to greater annotation accuracy by plurality voting when ETA is combined with homology methods. Figure 9a shows that in the PDB test set, BLAST+ETA plurality voting is always 100% accurate, even when only considering matches with less than 20% sequence identity (by comparison, the plurality voting accuracy of BLAST at less than 20% sequence identity is 88%). Likewise, plurality voting accuracy in the PSI set, while not 100%, is increased from 11% to 42%, relative to BLAST alone (Figure 9c). Although this increase in specificity is a trade-off with sensitivity Figures 9(b) and 9(d), these data confirm that ETA increases the predictive value of homology-based function annotations, particularly when there are no close homologs.

### Comparison with other template annotation methods

To further benchmark these results, we compared ETA to two of the template-based methods of ProFunc, since they also seek to find local geometric similarity between templates and protein structures. ProFunc offers four template-based methods of functional annotation, differing primarily in the source of their templates: ProFunc's Enzyme Active Site ("PEAS"), DNA-binding, ligand-binding, and reverse templates [22]. We first performed the comparison with the individual method used in ProFunc that most closely matches our own, PEAS. This method is both conceptually and methodologically similar to our own, with the major difference being the source of the input used to build the motifs: PEAS uses the available literature on catalytic sites, which is highly desirable for accuracy but due to the current paucity of such literature is much less so for usability, while ETA instead predicts functional residues by the well-known and experimentally-validated ET approach, which we hoped would provide similar levels of accuracy and much greater availability. To test this, we submitted each of the proteins in our PDB and PSI sets to the ProFunc server, whereupon PEAS annotations were taken as either the result of a plurality voting procedure, or as the top-scoring PEAS match.

ETA provides nearly four times as many predictions as PEAS and achieves an overall prediction accuracy that is 10% to 34% greater, as shown in Table 1. If only remote homologs with less than 20% sequence identity are allowed to match, ETA provides three times more true top predictions with the same level of accuracy as PEAS. PEAS builds templates from the available literature on known catalytic sites. This limited it to only 189 templates at the time of this analysis (spring 2007), and highlights the advantage of computing templates from evolutionary and structural considerations rather than relying on sparse experimental data.

Even when PEAS templates are available, ETA performs as well, or better. When PEAS templates predict a most-likely function, they achieve at most 71% accuracy, while in these cases ETA achieves 100% accuracy in the PDB set and only provides an incorrect prediction for a single template in the PSI set, for a minimum accuracy of 83%, or 74% at less than 20% sequence identity. For example in the case of glucose dehydrogenase (PDB 1SPX, chain A, EC 1.1.1), PEAS identifies a single match to a trihydroxynaphthalene reductase (PDB 1YBV, chain A, EC 1.3.1) with 27% sequence identity and a score of 358.094. ETA, however, finds 24 matches to the correct function (and none to a different function), 23 of which have less than 30% sequence identity. In another example, ubiquitin carboxyl-terminal hydrolase (PDB 1VJV, chain A, EC 3.1.2), PEAS identifies a single match to the sulfhydryl proteinase Papain (PDB 9PAP, EC 3.4.22, cysteine endopeptidase) with 17% sequence identity and a low score of 137.805. ETA finds a single match to a ubp-family deubiquitinating enzyme (PDB 1NBF, chain E, EC 3.1.2) with 20% sequence identity, which is the correct function.

Next we compared ETA with ProFunc's reverse-template approach ("PRT"), which like ETA also bypasses the need for experimental knowledge of the catalytic mechanism, although its method of doing so is quite different. Instead of defining a single template based on the available functional evidence, it instead tries several likely possibilities and scores the resulting large set of matches to identify likely functions [41]. Here the methodological differences and especially the level of access provided to this method by the ProFunc web server made such a comparison more difficult. First, only 5 of perhaps 20 significant matches are reported by ProFunc, leaving many of the matches with low sequence identity below the reporting cutoff. Thus, we were not able to perform the comparison with low sequence identity matches as we did for PEAS (the low number of hits by PEAS allows it to avoid this restriction). Second, the larger number of hits also exaggerated concerns about redundancy between matches, so we restricted our consideration to those in the PDB90 (this was not done in PEAS, as only 2 proteins contributed more than one match). Self-matches (matches sharing the same PDB code as the query) were also removed in all three methods.

Similar to PEAS, PRT annotations were taken as either the result of a plurality voting procedure, or as the top-scoring PRT match. Unlike PEAS, however, PRT sometimes found matches to non-enzymes, and these cases were counted as false predictions in the scoring method, although like ETA they did not contribute a vote in the EC plurality voting method. Overall, PRT identified a comparable number of matches to the other individual methods, but strikingly, only 25% of these were to the correct function (PPV = 25%, whereas that of ETA is 2.5-fold higher at 62%). These matches are contributed by all 49 proteins (no other method compared herein found predictions for all proteins), but only 30 proteins contribute a true match. Using the plurality voting procedure, 30 proteins have a vote winner, 28 of which are correct. This high level of accuracy and availability of predictions is only slightly lower than that of ETA. The scoring method increases the availability of predictions, as every protein has a top-scoring match, but this match is correct in only 43% of cases, thus decreasing prediction accuracy to less than half that provided by any other method except PEAS. This suggests that the results provided by PRT are more likely to be true when multiple hits are found to the same function. Even so, only 21 proteins with at least one true match were found out of 49 with matches (43%). The proportion of proteins contributing at least one true match out of the total that contribute matches is quite low in comparison to the other methods in Table 1. For example, even the use of plurality voting on PRT's matches yielded only 30 proteins with one true match out of 49 with matches (61%), whereas ETA finds 33 of 39 with matches (85%) and BLAST finds 39 of 44 (89%).

While the prediction accuracy and availability of PRT and ETA is similar in the PDB set (although the proportion of proteins with at least one true match is much lower), PRT performs better in the PSI set. Overall PRT identified 75 matches, 44 of which are correct (59% PPV, in comparison to ETA's 57%). Here, only 42 proteins contributed matches, but again this is a higher number than any other method, although only 30 provide a correct match. As in the PDB set, this proportion is lower than any of the other methods (PRT, 71%; ETA, 79%; BLAST, 84%). Using plurality voting on these matches, however, yields the highest accuracy of any individual method, with 26 correct votes out of 29 total (90% accuracy, 59% availability), compared to ETA's 22 correct votes out of 28 (79% accuracy, 57% availability). Again though, using the function of the top-scoring hit only provides 64% accuracy, the lowest of any method in Table 2, with 86% availability.

These results indicate that while PRT finds many false matches (as do all of the individual methods), and while the top-scoring hit is only correct about half of the time in the PDB and PSI sets, using plurality voting brings its accuracy to a level comparable with other methods in the PDB set and even exceeding that of the other methods in the PSI set, while maintaining nearly the same availability. ETA, in comparison, has slightly higher voting accuracy and availability in the PDB set, and much higher PPV and proportion of proteins with at least one true match. In the PSI set, ETA has lower voting accuracy, comparable voting availability and PPV, and again a much higher proportion of proteins with at least one true match. These results are remarkable considering that ETA uses but a single template whereas PRT uses many.

### Sensitivity Limitations

ETA's failure to match and annotate convergent proteins [67] is worth noting. It may have arisen for many reasons. First, examples may be lacking from the dataset. But, an ETA search on a serine protease (query PDB 1SPX, EC 3.4.21) produced 21 hits to other serine proteases, none of which was to a protein with a different fold than the query. Thus the templates are fold-specific. One reason may be that they are too large, as choosing six C_α _atoms from a surface trace cluster is less precise than and may not be as general as specific atoms from the classic "catalytic triad" of serine proteases. Or perhaps the ETA templates, which are rigid, cannot overcome tolerated conformational differences. Finally, 3D templates would be unlikely to identify wholesale rearrangements of the catalytic residues or changes in the mechanism itself [51]. In the future, multiple geometric representations of smaller templates may improve sensitivity across folds.

ETA also failed to identify some homologs with identical functions. These arose for one of three reasons. First, the amino acid type allowed to match at each template position was occasionally incomplete because representative sequences were missing from or too infrequent in the alignment. Such infrequent variations may be genuine allowable substitutions, such as the case of the nitrile hydratase (PDB 2AHJ, chain A) and the glutathione s-transferase (PDB 1E6B, chain A); a similar event occurs in the case of the cellulase (PDB 1CEN) where none of the sequences chosen for inclusion in the multiple sequence alignment contained the appropriate substitution, which is found in just one of the BLAST matches.

Second, the SVM does not perfectly separate relevant from random matches. Some matches fall on the wrong side of the separating hyperplane, as happened for a glutathione s-transferase (PDB 1F2E, chain A) – a problem that may be reduced with additional training data or features.

Third, the problem occasionally lies in confused annotation of multi-domain proteins, when ETA incorrectly treats the entire protein as having one function. If it then picks template residues from the domain that is missing annotation, as happened in the case of the topoisomerase (PDB 1EJ9, chain A), an error ensues.

Finally, to further understand false negatives missed by both ETA and BLAST, we compared functional sites as defined by both SITE [4] or CSA [52] but found no similarities that would have been recognized by ETA or BLAST. Such remote homologs and analogs may perform the same function via a different mechanism or geometry [51], which would be very difficult if not impossible for 3D template methods to find.

### Specificity Limitations

False positives are also a concern. These arose often due to missing, partial, or wrong EC annotations. For example, two matches to a Rieske iron-sulfur protein template (PDB 1RIE) lack EC annotation in the PDB and a third had an EC number different at the second digit. Yet all three, considered false, in fact have identical Gene Ontology (GO) [68] biochemical function annotation with the query ("ubiquinol-cytochrome-c reductase activity"). Likewise, two matches to a cAMP-dependent protein kinase template (PDB 1RGS) have no EC annotation but they share the same GO biochemical function annotation ("cAMP-dependent protein kinase regulator activity"). Similarly, a match to a beta-lactamase template (PDB 1K55, chain A) without EC annotation in the PDB also shares GO biochemical annotation ("penicillin-binding, beta-lactamase activity") with the query. Finally, in one instance, a match to a glutathione transferase (PDB 6GSV, chain A) template failed to be recognized as correct because the EC annotation of the match (PDB 1HNL) was not extracted from its non-standard PDB file (released Dec. 22, 1994). These examples taken from the training set suggest that at least 7 of 8 matches classified as false positives are in fact correct.

## Conclusion

To address a key limitation of 3D templates to annotate enzyme function – the sparseness of available experimental data to define templates [56], this study tested the hypothesis that evolutionary information could be used instead. Evolutionary data are often plentiful, and easily analyzed to identify key functional sites and residues with which to search for functional similarities among proteins. Thus, evolution-based 3D template annotation could be attempted in any protein with known structure and sufficiently diverse sequence homologs.

The automated ETA method was implemented to pick six of the most evolutionarily important residues from the surface of a protein, represent them by their C_α _atoms, identify their relevant matches in the PDB and to pick the function with a plurality of matches. The most stringent tests, on 49 PSI proteins, show that ETA narrows the list of likely functions to just a few possibilities and correctly identifies the single most likely function in 79% of cases. Although these predictions are currently limited to homologs, they are not trivial since they involve predictions on proteins with low sequence identity. Thus ETA is complementary to homology-based annotation: in the PSI set, combining these approaches raises voting accuracy by 20% relative to BLAST alone. Significantly, this improvement is even greater in the region of low sequence identity – precisely where homology methods are known to be less accurate.

These results prove the hypothesis, extend the range of application of 3D template functional annotation to a majority of enzymes with little or no information on their catalytic activity, and highlight the central role that evolutionary information can play at every step of function prediction: template definition, geometric matching, and filtering matches based on their ET ranks. The results also show that despite the fairly low atomic resolution of 3D templates, limited to C_α_-only representation, the evolutionary information provided by ET captures some of the key determinants of catalysis, and leads to a general method to build 3D templates and improves the accuracy of functional annotation.

Future directions should include more refined descriptions of side chain atoms; taking into account experimental information when available; exploring new matching strategies to allow larger-scale application; and integrating functional prediction with alternative methods of annotations. The approach could be extended to non-enzymes, using ET ranks to suggest 3D templates for co-factors, small ligands, or macromolecular interactions, and using GO annotations [68] for functional predictions. This approach may also have application beyond function annotation, for example fold-specific rather than function-specific residues could be used to annotate SCOP fold classifications [69,70]. For now, this fully automated functional annotation pipeline (to be available at our web site [71]) increases the accuracy of enzyme annotations for structural genomics, often narrowing experimental confirmation to one or just a few likely functions for which to assay.

## Methods

### Datasets

The training set was chosen to be diverse at the levels of sequence, structure, and function. It contains 53 diverse enzymes with less than 25% sequence identity to one another, representing 33 4-digit EC numbers and 13 3-digit EC numbers. The first test set, the PDB set, includes 49 enzymes with known function randomly chosen to be representative of the PDB. The second, the PSI set, was built similarly with structures from the PSI to assess performance in structural genomics proteins. Both originally contained 50 proteins, but one from each was also found in the training set (with the same PDB code) and removed. The remaining proteins in the PDB and PSI sets have no more than 38% and 44% sequence identity to any protein in the training set, respectively. These proteins had available functional annotations in the form of complete 4-digit EC numbers. The PDB code, EC annotation, SCOP class, SCOP fold, and functions of each protein in these three datasets are available in the supplementary tables [see Additional file 1, Tables S2–S4]. Templates were searched against one of two target sets of annotated proteins. For the optimization experiments (Figure 2), matches were searched against 13,600 chains from the 2002 PDB. This set includes only a single chain for proteins with multiple chains due to crystallographic symmetry. Mutants, ionically perturbed structures, and small peptide fragments were manually identified and removed. For all other experiments, templates were searched against the 2004 PDB-SELECT-90, (PDB90) [72,73], a representative, non-redundant subset of the PDB with less than 90% sequence identity to one another (at the time the most computationally-intensive portion of this work was done, the 2006 version was not yet available). This dataset of 8,600 proteins decreases bias towards overrepresented proteins such as lysozyme, which lead to incorrect results from our statistical and machine learning filters in cases where such proteins represent more than 1% of the entire set of structures in the PDB, and also makes the computationally-intensive template matching step (see below) more practical. In the 2002 PDB and PDB90 sets, 5,200 and 2,800 proteins have full, unambiguous (only one) 4-digit EC annotation while 7,900 and 5,400 have none. Only 500 (<4%) and 400 (<5%) matches have partial or ambiguous (i.e. more than one) EC annotation. These matches were discarded and not counted as either true or false.

### 3D Template Creation

ET analyses were performed using an automated [55], real-valued [74] version of the ET algorithm [53]. The evolutionary importance and the alignment provided for ET will be used to create templates. The Template Picker starts with the best-ranking residues and then consider lower-ranking residues until the largest structural cluster (all residues having at least one non-hydrogen atom within 4 Å of another residue in the cluster) contains at least 10 residues, excluding those with solvent accessibility less than 2 as measured by DSSP [75] (a cutoff chosen by manual observation).

Next, template residues are chosen from the surface trace cluster according to one of the following heuristics. "ET Rank" picks the most evolutionarily important residues; "No Surface" eliminates the surface constraint; "Start Surface" starts with the most solvent-accessible residue and then picks nearby evolutionarily important ones; "Conservation" favors residues with the least amino acid variation in the alignment; "Compact" starts at the centroid of the cluster and picks nearby residues; "Cleft" starts at the least solvent-accessible residue and then picks nearby residues that are relatively inaccessible to solvent; and "Solvent Accessibility" picks the most solvent accessible residues, regardless of their evolutionary importance. All heuristics break ties by choosing residues closest to the average position of the centroids of the current template residues and the trace cluster.

These residues are represented as a set of geometric points labeled with their evolutionary importance and amino acid types that appear more than once in the corresponding column of the multiple sequence alignment. Single-point methods choose points according to one of the following heuristics. "C_α _" and "C_β _" use the C_α _or C_β _atoms of the residues; "Average C_α_-C_β _" uses a point between the C_α _and C_β _atoms; and "Average All" uses the centroid of the amino acid (excluding hydrogen atoms). Similarly, multiple-point methods use the following heuristics: "C_α_+Average Sidechain" uses the C_α _atom and the centroid of the sidechain; "C_α_+C_β _" uses both the C_α _atom and the C_β _atom; and "All Atoms" uses a set of points representing each non-hydrogen atom in the amino acid.

### Template Matching

To find matches to a template in a set of target structures, we applied Match Augmentation, a geometric pattern matching tool that uses the evolutionary importance of each residue and the amino acid variability information at those positions [58,59,76]. MA matches a query template to a target structure in two stages: seed matching first identifies several low RMSD matches to the template's three highest-ranked residues; augmentation then iteratively matches remaining template residues in order of their ET rank. MA then outputs the lowest RMSD match if one is found. This enables MA to search the PDB for matches to a typical template in about 40 minutes on a single processor. ETA then computes the statistical significance (p-value) of a match using a nonparametric density estimate of the distribution of match RMSDs to a target set to obtain a list of significant matches.

### Evaluation of Matches

EC annotations [60] are those reported in the PDB. We define a true functional match as either exact agreement of all 4 digits of the hierarchical EC number (optimization experiments) or those that share the first 3 digits (all other experiments, unless otherwise stated), excluding matches to the same PDB code as the query. Matches to proteins without EC numbers are conservatively classified as false matches in calculating sensitivity and positive predictive value, but ignored completely in plurality voting. PPV and sensitivity measurements are calculated using all matches, while accuracy and availability refer to the single most likely function predicted by a plurality of matches. Negative predictive value and specificity are not reported because they always exceeded 99% due to the ability of the SVM and statistical filters to remove the vast majority of matches to proteins of different functions.

### Machine Learning Filtering of Matches

Once MA and the statistical filter identify the matches with significant chemical and geometric similarity, an SVM identifies likely functional matches using a 7-dimensional vector representing the RMSD of the match (one dimension) and the evolutionary importance of each of the target residues (6 dimensions, one for each of the 6 matched residues). The former is obtained from the MA program described above for each match passing through the statistical filter, and the latter is calculated as simply the sorted normalized ET rank of each target residue in the optimization experiments (for the template size experiment, the dimensionality of the vector was adjusted according to the number of residues in the template), and in all other experiments as the sorted absolute value of the difference in ET rank between each of the 6 matching template and target residues, with the latter method providing a modest improvement over the former. SVMs were implemented using the Spider package for MATLAB [77] using default settings with a balanced ridge parameter (calculated as the difference between the proportions of matches with the same or different functions in the training data) and an RBF kernel with σ = 0.5 (0.5 was found to provide the largest increase in SVM PPV over a linear kernel without decreasing sensitivity amongst a range of possible parameter values: .01, .05, .1, .25, .5, 1, 2, 5). To avoid bias in the optimization experiments, predictions on each training set protein were made by an SVM trained without representatives of its 4-digit EC number (i.e., 33 SVMs were made, each excluding one of the 33 functions in the dataset).

### BLAST and PSI-BLAST

BLAST and PSI-BLAST searches were performed against the sequences in the 2004 PDB-90. Matches with an e-value of 0.05 or better were selected. For PSI-BLAST, 2 iterations were performed and sequences with an e-value below the cutoff in either iteration were selected.

## Abbreviations

PSI – Protein Structure Initiative

ET – Evolutionary Trace

ETA – Evolutionary Trace Annotation

PDB – Protein structure DataBank

MA – Match Augmentation

RMSD – least Root Mean Squared Deviation

SVM – Support Vector Machine

EC – Enzyme Commission

PPV – Positive Predictive Value

CSA – Catalytic Site Atlas

PEAS – Profunc's Enzyme Active Site templates

PRT – Profunc's Reverse-Template approach

GO – Gene Ontology

## Authors' contributions

The code was primarily developed as follows: template heuristics and overall automation (DMK), geometric matching (BYC, LEK), geometric statistics (VYF, MK), support vector machine (RMW). The data sets were collected and analyzed by DMK, RMW, AML, and SE. The strategy and research design were led by DMK and OL with contributions from RMW and all authors. The manuscript was jointly written by DMK, RMW, and OL, with inputs from SE and all of the other authors.

## Supplementary Material

Additional file 1 Supplementary material (Supplementary Tables S1–4) is provided as an HTML-formatted webpage. Supplementary Table S1: PSI Set voting as sequence identity threshold of matched target pairs decreases. Supplementary Table S2: Training Set. Supplementary Table S3: PDB Set. Supplementary Table S4: PSI Set. Click here for file
