# Supplementary material for: Prediction of enzyme function based on 3D templates of evolutionarily important amino acids
Source: BMC Bioinformatics. 2008 Jan 11;9:17. doi: 10.1186/1471-2105-9-17 (PMC2219985; doi:10.1186/1471-2105-9-17)
Supplement: Additional file 1 — Supplementary material (Supplementary Tables S1–4) is provided as an HTML-formatted webpage. Supplementary Table S1: PSI Set voting as sequence identity threshold of matched target pairs decreases. Supplementary Table S2: Training Set. Supplementary Table S3: PDB Set. Supplementary Table S4: PSI Set. [file 1471-2105-9-17-S1.HTM]

### Supplementary Table S1: PSI Set voting as sequence identity threshold of matched target pairs decreases.

|  |  |  |  |
| --- | --- | --- | --- |
| **Sequence Identity Cutoff** | **Correct Votes** | **Incorrect Votes** | **No Vote Winners** |
| 100 | 22 | 6 | 21 |
| 80 | 22 | 6 | 21 |
| 60 | 22 | 5 | 22 |
| 40 | 20 | 5 | 24 |
| 35 | 20 | 5 | 24 |
| 30 | 20 | 5 | 24 |
| 25 | 20 | 6 | 23 |
| 20 | 19 | 6 | 24 |
| 15 | 19 | 7 | 23 |
| 10 | 21 | 13 | 15 |
| 5 | 21 | 13 | 15 |

 

### Supplementary Table S2: Training Set.

|  |  |  |  |  |
| --- | --- | --- | --- | --- |
| **Protein** | **E.C. #** | **SCOP Class** | **SCOP Fold** | **Function** |
| 1rie\_ | 1.10.2.2 | b | ISP domain | RIESKE IRON-SULFUR PROTEIN |
| 1mgtA | 2.1.1.63 | a,a/b | DNA/RNA-binding 3-helical bundle Ribonuclease H-like motif | O6-METHYLGUANINE-DNA METHYLTRANSFERASE |
| 1eg2A | 2.1.1.72 | a/b | S-adenosyl-L-methionine-dependent methyltransferases | MODIFICATION METHYLASE RSRI |
| 7mhtA | 2.1.1.73 | a/b | S-adenosyl-L-methionine-dependent methyltransferases | CYTOSINE-SPECIFIC METHYLTRANSFERASE HHAI |
| 1jg1A | 2.1.1.77 | a/b | S-adenosyl-L-methionine-dependent methyltransferases | PROTEIN-L-ISOASPARTATE O-METHYLTRANSFERASE |
| 1kpgA | 2.1.1.79 | a/b | S-adenosyl-L-methionine-dependent methyltransferases | CYCLOPROPANE-FATTY-ACYL-PHOSPHOLIPID SYNTHASE 1 |
| 1inlB | 2.5.1.16 | a/b | S-adenosyl-L-methionine-dependent methyltransferases | SPERMIDINE SYNTHASE |
| 1g64B | 2.5.1.17 | a/b | P-loop containing nucleoside triphosphate hydrolases | COB(I)ALAMIN ADENOSYLTRANSFERASE |
| 1e6bA | 2.5.1.18 | a,a/b | Glutathione S-transferase (GST), C-terminal domain Thioredo | GLUTATHIONE S-TRANSFERASE |
| 1f2eA | 2.5.1.18 | a,a/b | Glutathione S-transferase (GST), C-terminal domain Thioredo | GLUTATHIONE S-TRANSFERASE |
| 1gsfA | 2.5.1.18 | a,a/b | Glutathione S-transferase (GST), C-terminal domain Thioredo | GLUTATHIONE TRANSFERASE A1-1 |
| 6gsvA | 2.5.1.18 | a,a/b | Glutathione S-transferase (GST), C-terminal domain Thioredo | MU CLASS GLUTATHIONE S-TRANSFERASE OF ISOENZYME 3-3 |
| 1bdyA | 2.7.1.37 | b | PH domain-like | PROTEIN KINASE C |
| 1iasA | 2.7.1.37 | small | Cystine-knot cytokines | TGF-BETA RECEPTOR TYPE I |
| 1qf8A | 2.7.1.37 | b | Galactose-binding domain-like | CASEIN KINASE II |
| 1rgs\_ | 2.7.1.37 | b | C2 domain-like | CAMP DEPENDENT PROTEIN KINASE |
| 1b55B | 2.7.1.112 | a+b | Protein kinase-like (PK-like) | TYROSINE-PROTEIN KINASE BTK |
| 1fltW | 2.7.1.112 | small | Rubredoxin-like | VASCULAR ENDOTHELIAL GROWTH FACTOR |
| 1nukA | 2.7.1.112 |  |  | TYROSINE-PROTEIN KINASE RECEPTOR EPH |
| 1nksA | 2.7.4.3 | a/b | P-loop containing nucleoside triphosphate hydrolases | ADENYLATE KINASE |
| 1nskL | 2.7.4.6 | a+b | Ferredoxin-like | NUCLEOSIDE DIPHOSPHATE KINASE |
| 1jxiA | 2.7.4.7 | a/b | Ribokinase-like | PHOSPHOMETHYLPYRIMIDINE KINASE |
| 1c25\_ | 3.1.3.48 | a/b | Rhodanese/Cell cycle control phosphatase | CDC25A |
| 1gwz\_ | 3.1.3.48 | a/b | (Phosphotyrosine protein) phosphatases II | SHP-1 |
| 1j4xA | 3.1.3.48 | a/b | (Phosphotyrosine protein) phosphatases II | DUAL SPECIFICITY PROTEIN PHOSPHATASE 3 |
| 1cen\_ | 3.2.1.4 | b,a/b | WW domain-like TIM beta/alpha-barrel FKBP-like | CELLULASE CELC |
| 1edg\_ | 3.2.1.4 | b | Single-stranded right-handed beta-helix | ENDOGLUCANASE A |
| 2nlrA | 3.2.1.4 | a+b | Lysozyme-like | ENDOGLUCANASE |
| 7a3hA | 3.2.1.4 | a+b | Lysozyme-like | ENDOGLUCANASE |
| 1i8a\_ | 3.2.1.8 | b | 6-bladed beta-propeller | ENDO-1,4-BETA-XYLANASE A |
| 1ukrA | 3.2.1.8 | a/b | TIM beta/alpha-barrel | ENDO-1,4-B-XYLANASE I |
| 1goiA | 3.2.1.14 | a/b | TIM beta/alpha-barrel | CHITINASE B |
| 1czfA | 3.2.1.15 | b | Concanavalin A-like lectins/glucanases | POLYGALACTURONASE II |
| 1d9uA | 3.2.1.17 | a/b | TIM beta/alpha-barrel | BACTERIOPHAGE LAMBDA LYSOZYME |
| 3lzt\_ | 3.2.1.17 |  |  | LYSOZYME |
| 1f8eA | 3.2.1.18 | b | Concanavalin A-like lectins/glucanases | NEURAMINIDASE |
| 1fr6A | 3.5.2.6 | mult | beta-lactamase/transpeptidase-like | BETA-LACTAMASE |
| 1k55A | 3.5.2.6 | mult | beta-lactamase/transpeptidase-like | BETA LACTAMASE OXA-10 |
| 1pioA | 3.5.2.6 | mult | beta-lactamase/transpeptidase-like | BETA-LACTAMASE |
| 1juk\_ | 4.1.1.48 | a/b | TIM beta/alpha-barrel | INDOLE-3-GLYCEROL PHOSPHATE SYNTHASE |
| 1ayl\_ | 4.1.1.49 |  |  | PHOSPHOENOLPYRUVATE CARBOXYKINASE |
| 1jl0B | 4.1.1.50 | a+b | S-adenosylmethionine decarboxylase | S-ADENOSYLMETHIONINE DECARBOXYLASE PROENZYME |
| 2ahjA | 4.2.1.84 | a+b | Nitrile hydratase alpha chain | NITRILE HYDRATASE |
| 1dcpA | 4.2.1.96 | a+b | DCoH-like | DCOH |
| 1ep0A | 5.1.3.13 | b | Double-stranded beta-helix | DTDP-6-DEOXY-D-XYLO-4-HEXULOSE 3,5-EPIMERASE |
| 1eq2H | 5.1.3.20 | a/b | NAD(P)-binding Rossmann-fold domains | ADP-L-GLYCERO-D-MANNOHEPTOSE 6-EPIMERASE |
| 1a41\_ | 5.99.1.2 | a+b | DNA breaking-rejoining enzymes | TOPOISOMERASE I |
| 1cy4A | 5.99.1.2 | mult | Prokaryotic type I DNA topoisomerase | DNA TOPOISOMERASE I |
| 1ej9A | 5.99.1.2 | a+b,mult | DNA breaking-rejoining enzymes Eukaryotic DNA topoisomerase | DNA TOPOISOMERASE I |
| 1vcc\_ | 5.99.1.2 | a+b | DNA topoisomerase I domain | DNA TOPOISOMERASE I |
| 1fpmA | 6.3.4.3 | a/b | P-loop containing nucleoside triphosphate hydrolases | FORMATE--TETRAHYDROFOLATE LIGASE |
| 1dj3A | 6.3.4.4 | a/b | P-loop containing nucleoside triphosphate hydrolases | ADENYLOSUCCINATE SYNTHETASE |
| 1k92A | 6.3.4.5 | a/b,a+b | Adenine nucleotide alpha hydrolase-like Argininosuccinate s | ARGININOSUCCINATE SYNTHASE |

                                               

### Supplementary Table S3: PDB Set.

|  |  |  |  |  |
| --- | --- | --- | --- | --- |
| **Protein** | **E.C. #** | **SCOP Class** | **SCOP Fold** | **Function** |
| 1hdoA | 1.3.1.24 | a/b | NAD(P)-binding Rossmann-fold domains | BILIVERDIN IX BETA REDUCTASE |
| 1b26A | 1.4.1.3 | a/b | NAD(P)-binding Rossmann-fold domains | GLUTAMATE DEHYDROGENASE |
| 1kmvA | 1.5.1.3 | a/b | Dihydrofolate reductases | DIHYDROFOLATE REDUCTASES, EUKARYOTIC TYPE |
| 1l1dA | 1.8.4.5 | b | Mss4-like | C-TERMINAL MSRB DOMAIN OF PEPTIDE METHIONINE SULFOXIDE REDUCTASE PILB |
| 1v54J | 1.9.3.1 | mem | Single transmembrane helix | MITOCHONDRIAL CYTOCHROME C OXIDASE SUBUNIT VIIA |
| 1eb7A | 1.11.1.5 | a | Cytochrome c | DI-HEME CYTOCHROME C PEROXIDASE |
| 1lycA | 1.11.1.7 | a | Heme-dependent peroxidases | FUNGAL PEROXIDASE (LIGNINASE) |
| 1hqi\_ | 1.14.13.7 | a+b | Monooxygenase (hydroxylase) regulatory protein | PHENOL HYDROXYLASE P2 PROTEIN |
| 1oykA | 1.14.99.3 | a | Heme oxygenase-like | HEME OXYGENASE-1 (HO-1) |
| 1m1nA | 1.18.6.1 | a/b | Chelatase-like | NITROGENASE IRON-MOLYBDENUM PROTEIN, ALPHA CHAIN |
| 1orhA | 2.1.1.125 | a/b | S-adenosyl-L-methionine-dependent methyltransferases | PROTEIN ARGININE N-METHYLTRANSFERASE 1, PRMT1 |
| 1ib1A | 2.3.1.87 | a | alpha-alpha superhelix | 14-3-3 PROTEIN, ZETA ISOFORM |
| 1o7qA | 2.4.1.151 | a/b | Nucleotide-diphospho-sugar transferases | ALPHA-1,3-GALACTOSYLTRANSFERASE CATALYTIC DOMAIN |
| 1nh7A | 2.4.2.17 | a/b | Periplasmic binding protein-like II | ATP PHOSPHORIBOSYLTRANSFERASE (ATP-PRTASE, HISG), CATALYTIC DOMAIN |
| 1fsgA | 2.4.2.8 | a/b | PRTase-like | HYPOXANTHINE-GUANINE-XANTHINE PRTASE |
| 6pfkA | 2.7.1.11 | a/b | Phosphofructokinase | ATP-DEPENDENT PHOSPHOFRUCTOKINASE |
| 1r79A | 2.7.1.107 | small | Cysteine-rich domain | DIACYLGLYCEROL KINASE DELTA |
| 1bx4A | 2.7.1.20 | a/b | Ribokinase-like | ADENOSINE KINASE |
| 1rdqE | 2.7.1.37 | a+b | Protein kinase-like (PK-like) | CAMP-DEPENDENT PK, CATALYTIC SUBUNIT |
| 1pdo\_ | 2.7.1.69 | a/b | IIA domain of mannose transporter, IIA-Man | IA DOMAIN OF MANNOSE TRANSPORTER, IIA-MAN |
| 1m15A | 2.7.3.3 | a | Guanido kinase N-terminal domain | ARGININE KINASE, N-DOMAIN |
| 1jxiA | 2.7.4.7 | a/b | Ribokinase-like | HMP-PHOSPHATE KINASE, THID |
| 1ef4A | 2.7.7.6 | a | DNA/RNA-binding 3-helical bundle | RNA POLYMERASE SUBUNIT RPB10 |
| 1p16A | 2.7.7.50 | a+b | ATP-grasp | MRNA CAPPING ENZYME ALPHA SUBUNIT |
| 1f7lA | 2.7.8.7 | a+b | 4'-phosphopantetheinyl transferase | HOLO-(ACYL CARRIER PROTEIN) SYNTHASE ACPS |
| 1okgA | 2.8.1.2 | a+b | FKBP-like | 3-MERCAPTOPYRUVATE SULFURTRANSFERASE |
| 1ksjB | 3.1.4.17 | b | Immunoglobulin-like beta-sandwich | GMP-PDE DELTA |
| 1jl1A | 3.1.26.4 | a/b | Ribonuclease H-like motif | RNASE H (RNASE HI) |
| 1i0vA | 3.1.27.3 | a+b | Microbial ribonucleases | RNASE T1 |
| 1hx0A | 3.2.1.1 | a/b | TIM beta/alpha-barrel | ANIMAL ALPHA-AMYLASE |
| 1ks8A | 3.2.1.4 | a | alpha/alpha toroid | ENDO-B-1,4-GLUCANASE |
| 3sil\_ | 3.2.1.18 | b | 6-bladed beta-propeller | SALMONELLA SIALIDASE |
| 1es5A | 3.4.16.4 | mult | beta-lactamase/transpeptidase-like | D-ALA CARBOXYPEPTIDASE/TRANSPEPTIDASE |
| 1bcrA | 3.4.16.6 | a/b | alpha/beta-Hydrolases | SERINE CARBOXYPEPTIDASE II |
| 1pq7A | 3.4.21.4 | b | Trypsin-like serine proteases | TRYPSIN(OGEN) |
| 2hrvA | 3.4.22.29 | b | Trypsin-like serine proteases | 2A CYSTEINE PROTEINASE |
| 1gk9A | 3.5.1.11 | a+b | Ntn hydrolase-like | PENICILLIN ACYLASE |
| 1uf4A | 3.5.1.77 | a+b | Carbon-nitrogen hydrolase | N-CARBAMOYL-D-AMINOACID AMIDOHYDROLASE |
| 1sg3A | 3.5.3.4 | b | Galactose-binding domain-like | ALLANTOICASE |
| 1i40A | 3.6.1.1 | b | OB-fold | INORGANIC PYROPHOSPHATASE |
| 1opzA | 3.6.3.4 | a+b | Ferredoxin-like | POTENTIAL COPPER-TRANSLOCATING P-TYPE ATPASE COPA (YVGX) |
| 1hxgA | 4.1.99.7 | a | alpha/alpha toroid | 5-EPI-ARISTOLOCHENE SYNTHASE |
| 1iv3A | 4.6.1.12 | a+b | Bacillus chorismate mutase-like | 2C-METHYL-D-ERYTHRITOL 2,4-CYCLODIPHOSPHATE SYNTHASE ISPF |
| 1pinA | 5.2.1.8 | a+b | FKBP-like | MITOTIC ROTAMASE PIN1, DOMAIN 2 |
| 1dl3A | 5.3.1.24 | a/b | TIM beta/alpha-barrel | N-(5'PHOSPHORIBOSYL)ANTRANILATE ISOMERASE, PRAI |
| 1fztA | 5.4.2.1 | a/b | Phosphoglycerate mutase-like | PHOSPHOGLYCERATE MUTASE |
| 1n3lA | 6.1.1.1 | a/b | Adenine nucleotide alpha hydrolase-like | TYROSYL-TRNA SYNTHETASE (TYRRS) |
| 1pfvA | 6.1.1.10 | a/b | Adenine nucleotide alpha hydrolase-like | METHIONYL-TRNA SYNTHETASE (METRS) |
| 1sbqA | 6.3.3.2 | a/b | NagB/RpiA/CoA transferase-like | 5,10-METHENYLTETRAHYDROFOLATE SYNTHETASE HOMOLOG MPN348 |

                                               

### Supplementary Table S4: PSI Set.

|  |  |  |  |  |
| --- | --- | --- | --- | --- |
| **Protein** | **E.C. #** | **SCOP Class** | **SCOP Fold** | **Function** |
| 1z82A | 1.1.1.8 | - | not available | GLYCEROL-3-PHOSPHATE DEHYDROGENASE |
| 1nvtA | 1.1.1.25 | a/b | Aminoacid dehydrogenase-like, N-terminal domain | SHIKIMATE 5-DEHYDROGENASE AROE |
| 1zejA | 1.1.1.35 | - | not available | 3-HYDROXYACYL-COA DEHYDROGENASE |
| 1spxA | 1.1.1.47 | a/b | NAD(P)-binding Rossmann-fold domains | GLUCOSE DEHYDROGENASE |
| 1yb4A | 1.1.1.60 | - | not available | TARTRONIC SEMIALDEHYDE REDUCTASE |
| 2amfA | 1.2.1.5 | - | not available | 1-PYRROLINE-5-CARBOXYLATE REDUCTASE |
| 1txnA | 1.3.3.3 | - | not available | COPROPORPHYRINOGEN III OXIDASE |
| 1vllA | 1.4.1.1 | a/b | NAD(P)-binding Rossmann-fold domains | ARCHAEAL ALANINE DEHYDROGENASE |
| 1ooeA | 1.6.99.7 | a/b | NAD(P)-binding Rossmann-fold domains | DIHYDROPTERIDIN REDUCTASE (PTERIDINE REDUCTASE) |
| 1nni1 | 1.7.1.6 | a/b | Flavodoxin-like | AZOBENZENE REDUCTASE |
| 1p91A | 2.1.1.51 | a/b | S-adenosyl-L-methionine-dependent methyltransferases | RRNA METHYLTRANSFERASE RLMA |
| 2ar0A | 2.1.1.72 | - | not available | TYPE I RESTRICTION ENZYME ECOKI M PROTEIN |
| 1vloA | 2.1.2.10 | a+b | Aminomethyltransferase folate-binding domain | GLYCINE CLEAVAGE SYSTEM T PROTEIN, GCVT |
| 1vlvA | 2.1.3.3 | a/b | ATC-like | ORNITHINE TRANSCARBAMOYLASE |
| 1vmiA | 2.3.1.8 | - | not available | PUTATIVE PHOSPHATE ACETYLTRANSFERASE |
| 1vlpA | 2.4.2.11 | a+b | alpha/beta-Hammerhead | NICOTINATE PHOSPHORIBOSYLTRANSFERASE, C-TERMINAL DOMAIN |
| 1yirA | 2.4.2.11 | - | not available | NICOTINATE PHOSPHORIBOSYLTRANSFERASE 2 |
| 1xi3A | 2.5.1.3 | a/b | TIM beta/alpha-barrel | THIAMIN PHOSPHATE SYNTHASE |
| 1iy9A | 2.5.1.16 | a/b | S-adenosyl-L-methionine-dependent methyltransferases | SPERMIDINE SYNTHASE |
| 1vjoA | 2.6.1.44 | a/b | PLP-dependent transferases | ALANINE-GLYOXYLATE AMINOTRANSFERASE |
| 1xx6A | 2.7.1.21 | - | not available | THYMIDINE KINASE |
| 1kagA | 2.7.1.71 | a/b | P-loop containing nucleoside triphosphate hydrolases | SHIKIMATE KINASE (AROK) |
| 2akoA | 2.7.2.11 | - | not available | GLUTAMATE 5-KINASE |
| 1ej2A | 2.7.7.1 | a/b | Adenine nucleotide alpha hydrolase-like | NICOTINAMIDE MONONUCLEOTIDE (NMN) ADENYLYLTRANSFERASE |
| 1r1dA | 3.1.1.1 | a/b | alpha/beta-Hydrolases | CARBOXYLESTERASE |
| 1vl1A | 3.1.1.31 | a/b | NagB/RpiA/CoA transferase-like | 6-PHOSPHOGLUCONOLACTONASE |
| 1vjvA | 3.1.2.15 | a+b | Cysteine proteinases | UBIQUITIN CARBOXYL-TERMINAL HYDROLASE 6 |
| 1xm8A | 3.1.2.6 | - | not available | GLYOXALASE II |
| 1vr0A | 3.1.3.71 | - | not available | PROBABLE 2-PHOSPHOSULFOLACTATE PHOSPHATASE |
| 1nxcA | 3.2.1.113 | a | alpha/alpha toroid | CLASS I ALPHA-1;2-MANNOSIDASE, CATALYTIC DOMAIN |
| 1o0xA | 3.4.11.18 | a+b | Creatinase/aminopeptidase | METHIONINE AMINOPEPTIDASE |
| 1o59A | 3.5.3.4 | b | Galactose-binding domain-like | ALLANTOICASE |
| 1xfkA | 3.5.3.8 | - | not available | FORMIMIDOYLGLUTAMASE |
| 1xsrA | 3.5.3.19 | - | not available | UREIDOGLYCOLATE HYDROLASE |
| 1yvwA | 3.6.1.31 | - | not available | PHOSPHORIBOSYL-ATP PYROPHOSPHATASE |
| 1tufA | 4.1.1.20 | a/b | TIM beta/alpha-barrel | DIAMINOPIMELATE DECARBOXYLASE LYSA |
| 1fi4A | 4.1.1.33 | a+b | Ribosomal protein S5 domain 2-like | MEVALONATE 5-DIPHOSPHATE DECARBOXYLASE |
| 1vpjA | 4.2.1.2 | - | not available | FUMARASE |
| 1kmjA | 4.4.1.16 | a/b | PLP-dependent transferases | NIFS-LIKE PROTEIN/SELENOCYSTEINE LYASE |
| 1kmkA | 4.4.1.16 | a/b | PLP-dependent transferases | NIFS-LIKE PROTEIN/SELENOCYSTEINE LYASE |
| 1y0eA | 5.1.3.9 | - | not available | PUTATIVE N-ACETYLMANNOSAMINE-6-PHOSPHATE 2-EPIMERASE |
| 1o8bA | 5.3.1.6 | a/b | NagB/RpiA/CoA transferase-like | D-RIBOSE-5-PHOSPHATE ISOMERASE (RPIA), CATALYTIC DOMAIN |
| 1x8mA | 5.3.1.17 | b | Double-stranded beta-helix | 5-KETO-4-DEOXYURONATE ISOMERASE KDUI |
| 1ou0A | 5.4.1.2 | a/b | Flavodoxin-like | PRECORRIN-8X METHYLMUTASE RELATED PROTEIN |
| 1m0tA | 6.3.2.3 | a+b | ATP-grasp | EUKARYOTIC GLUTATHIONE SYNTHETASE |
| 1kutA | 6.3.2.6 | a+b | SAICAR synthase-like | SAICAR SYNTHASE |
| 1pzvA | 6.3.2.19 | a+b | UBC-like | UBIQUITIN CONJUGATING ENZYME, UBC |
| 1vl2A | 6.3.4.5 | a+b | Argininosuccinate synthetase, C-terminal domain | ARGININOSUCCINATE SYNTHETASE |
| 1vkzA | 6.3.4.13 | a+b | ATP-grasp | GLYCINAMIDE RIBONUCLEOTIDE SYNTHETASE (GAR-SYN) |
